# Organizational resilience in healthcare: a review and descriptive narrative synthesis of approaches to resilience measurement and assessment in empirical studies

**DOI:** 10.1186/s12913-023-09242-9

**Published:** 2023-04-19

**Authors:** Agnieszka Ignatowicz, Carolyn Tarrant, Russell Mannion, Dena El-Sawy, Simon Conroy, Daniel Lasserson

**Affiliations:** 1 Institute of Applied Health Research, College of Medical and Dental Sciences, University of Birmingham, Birmingham, UK; 2 Department of Health Sciences, University of Leicester, Leicester, UK; 3 Russell Mannion, Health Services and Management Centre, College of Social Sciences, University of Birmingham, Birmingham, UK; 4 MRC Unit for Lifelong Health and Ageing, University College London, London, UK; 5 Warwick Medical School, University of Warwick, Coventry, UK

**Keywords:** Organisational resilience, Healthcare resilience, Approaches to resilience measurement, Resilience assessment, Resilience characteristics and indicators

## Abstract

**Background:**

The coronavirus pandemic has had a profound impact on organization and delivery of care. The challenges faced by healthcare organizations in dealing with the pandemic have intensified interest in the concept of resilience. While effort has gone into conceptualising resilience, there has been relatively little work on how to evaluate organizational resilience. This paper reports on an extensive review of approaches to resilience measurement and assessment in empirical healthcare studies, and examines their usefulness for researchers, policymakers and healthcare managers.

**Methods:**

Various databases (MEDLINE, EMBASE, PsycINFO, CINAHL (EBSCO host), Cochrane CENTRAL (Wiley), CDSR, Science Citation Index, and Social Science Citation Index) were searched from January 2000 to September 2021. We included quantitative, qualitative and modelling studies that focused on measuring or qualitatively assessing organizational resilience in a healthcare context. All studies were screened based on titles, abstracts and full text. For each approach, information on the format of measurement or assessment, method of data collection and analysis, and other relevant information were extracted. We classified the approaches to organizational resilience into five thematic areas of contrast: (1) type of shock; (2) stage of resilience; (3) included characteristics or indicators; (4) nature of output; and (5) purpose. The approaches were summarised narratively within these thematic areas.

**Results:**

Thirty-five studies met the inclusion criteria. We identified a lack of consensus on how to evaluate organizational resilience in healthcare, what should be measured or assessed and when, and using what resilience characteristic and indicators. The measurement and assessment approaches varied in scope, format, content and purpose. Approaches varied in terms of whether they were prospective (resilience pre-shock) or retrospective (during or post-shock), and the extent to which they addressed a pre-defined and shock-specific set of characteristics and indicators.

**Conclusion:**

A range of approaches with differing characteristics and indicators has been developed to evaluate organizational resilience in healthcare, and may be of value to researchers, policymakers and healthcare managers. The choice of an approach to use in practice should be determined by the type of shock, the purpose of the evaluation, the intended use of results, and the availability of data and resources.

**Supplementary Information:**

The online version contains supplementary material available at 10.1186/s12913-023-09242-9.

## Background

The coronavirus disease 2019 (Covid-19) pandemic has had a profound impact on the organization and delivery of acute care around the world, requiring the allocation and reorganization of resources to Covid-19 positive patients [1, 2]. In the early phases of the pandemic, the focus was on building capacity and providing supportive care to patients. However, as many countries have been tackling ongoing Covid-19 waves, there is also a strong imperative to determine how best to deal with backlogs and deliver “business as usual” alongside preparing to respond to future surges in demand created by current and possible future outbreaks. The challenge faced by healthcare organizations in dealing with Covid-19 is set against a backdrop of high demand for urgent healthcare, with periods of sustained high volumes of activity, for example, during the winter season. To meet this challenge, policymakers and researchers are increasingly turning to academic literature to understand what constitutes resilience in healthcare organisations and how this concept can be best understood and measured [3].

Resilience describes the intrinsic ability of a system to adjust its functioning prior to, during, or following changes and disturbances so that it is able to sustain required operations under both expected and unexpected conditions [4, 5]. Better understanding of how healthcare organisations anticipate, monitor, respond to, and learn from sudden events and everyday challenges has the potential to support better delivery of patient care through evidence-informed healthcare policy focussed on enhancing the response to current and future pandemics [3, 6, 7].

Despite the increased interest in the concept of resilience and its practical applications among researchers, policymakers and healthcare managers, there are relatively few studies focussing on measurement of resilience in organisations, and even fewer on the development of resilience measurement frameworks and indices for healthcare. Well-formulated and conceptually grounded approaches to measuring resilience are critical for identifying and learning from resilient organisations, supporting improvement, and assessing the impact of strategies to improve resilience on outcomes. As such, developing methods or metrics for measuring and monitoring resilience in healthcare is becoming a high priority [3, 8, 9], further intensified by the Covid-19 pandemic. It has been argued, however, that the lack of clarity on the definition and scope of the concept has resulted in difficulties in defining the appropriate measurement and assessment methods [7, 8]: there is still much confusion about what resilience means in a healthcare context, and more importantly, “what to measure, whom to measure, how often to measure, what methods to use, and at what scale” [10] (p.7). To measure resilience in healthcare, we need to know exactly what resilience is in a defined context, what factors contribute to it, and for what types of shocks [9, 11]. Resilience is specific to contexts (i.e., time, space and type of shock) and the nature of the resilient response will depend on the stage of the shock cycle (before, during or after the shock strikes) [12]. The ability to measure resilience depends on the analysis of these dimensions because they highlight the specific indicators and data that need to be collected [13].

Resilience can be conceptualised at different temporal and spatial levels in a system. Andersen et al.’s [14] model of adaptive capacity in healthcare describes: situated resilience, involving anticipation, adjustment and learning during care delivery; structural level resilience where the organisation delivers—“infrastructure planning and provision, organisational performance monitoring, emergency response planning and workforce planning” and systemic resilience, involving strategy and planning by government, policymakers and regulators. Empirical research has described the specific capacities and processes that enhance resilience at different levels within a healthcare system and across diverse healthcare contexts [4, 8, 15–17].

Most research into the measurement of resilience in healthcare has focused on conceptualising or measuring resilience at the macro-level of the health system, with indicators differing in the level at which data were collected [8]. Thomas et al.’s [7] rapid review of literature provides a list of metrics that can be used to assess health system resilience in relation to governance, financing, resource generation and service delivery, whereas Fleming et al.’s [18] systematic review explores different metrics and indicators used to assess health system resilience in response to shocks. Arguably, however, much of the work involved in responding to the demands of Covid-19, along with other chronic stressors such as winter pressures, is situated at the meso or organisational level. At this meso-level, organisational resilience reflects how hospitals or healthcare clinics monitor and plan for fluctuations in demand, cope with shocks or stressors, and adapt and learn. Organisations can demonstrate resilience in the face of challenges in many ways, from reconfiguring resources and reshaping relationships to optimizing processes and changing protocols and guidelines [19]. External and internal factors can contribute to organisational resilience [20, 21], but very few publications highlight important relationships and interactions between the different resilience levels in the evaluation process [15, 22]. Focusing on the evaluation of organizational resilience means considering the specific capacities and processes that enhance resilience within organizations: how these intersect with resilience practices at the frontline, and resilience across healthcare systems, is a broader and more challenging issue to address in the context of evaluation.

Existing literature reviews of efforts to measure organisational resilience beyond healthcare have identified a general lack of consensus in the conceptualisation and operationalisation of the concept [20, 23–25]. Several models and metrics have been developed, but no universally accepted approach for measuring or assessing organisational resilience exists. A range of scales have been developed, mainly from retrospective analysis of organisational performance [26]. These scales either measure a set of characteristics such as knowledge, learning, planning, agility, adaptively, robustness (e.g. [27–29]) or evaluate capacity to deal with shocks and stresses (e.g. [30, 31]), but their applicability to a healthcare context may be limited. Most recently, a review classified 30 papers that proposed tools or methods to measure organisational resilience into those that use the features of the organisation, those that use organisational outcomes, and those that focus on how the organization recovers from challenges [32].

Against this conceptual and empirical background, we conducted a systematic review of approaches to assess or measure organisational resilience in healthcare, in an attempt to delineate common approaches, methods and indicators. This review makes a significant contribution by providing evidence and practical lessons drawn from the published literature, to inform decision-making about approaches to resilience assessment and measurement in healthcare.

## Ethical approval

This review is part of a larger project funded by the National Institute for Health Research (NIHR) Policy Research Programme (PRP). The project focuses on how best to configure acute medical services to meet the needs of patients during peaks of COVID-19 infections and more generally, during periods of heightened demand for acute care. Ethical approval was not required for this component of the study as this was a systematic review of peer-reviewed journal articles.

## Methods

A protocol for the systematic review was registered with PROSPERO (registration number: CRD42021254780). Throughout this review, we followed the Preferred Reporting Items for Systematic Reviews and Meta-Analysis (PRISMA) statement [33].

### Search strategy

In collaboration with an experienced information specialist, we searched MEDLINE and MEDLINE In Process, EMBASE, PsycINFO and HMIC (all via the Ovid platform), CINAHL (EBSCO host), Cochrane CENTRAL (Wiley), CDSR, Science Citation Index, Social Science Citation Index and Conference Proceedings Citation Index (Web of Science). In addition to these, biomedical databases grey literature (including Joanna Briggs Institute, ClinicalTrials.gov and Open Grey) were also searched. We used systematic search strategies (details of an example of search strategy and sources used are provided in Additional file 1). The main search was conducted in September 2020, and was limited to articles published since January 2000, in English. Search results from all databases were combined and deduplication performed using Endnote. The references of each included paper were also searched for relevant studies. We updated this search in MEDLINE and EMBASE in September 2021 to capture any new and relevant studies that were published since the Covid-19 pandemic began.

### Eligibility criteria

We used the following inclusion criteria to select papers for this review: papers reporting studies that (1) were specific to *organisational* resilience in *healthcare*; and (2) involved *measuring resilience* (e.g., through checklists or surveys) or *assessing resilience* (e.g., using qualitative assessment methods). We did not specify a specific definition of resilience, to be comprehensive in our review, and included studies from various academic fields such as national disaster preparedness and emergency management. Studies that reported on the use of an existing framework, model, checklist, or tool for assessment or measurement of resilience in healthcare organisation/s were included, as were studies that developed an approach for assessing or measuring resilience and piloted or tested it.

Non-English language publications, papers without full text or those where the full text was not available, were excluded. Studies that were related to the micro level (resilience of healthcare staff or teams), or macro level (national or regional health systems), were excluded, as were studies that only reported on developing a measure without applying or testing it. Systematic reviews and meta-analysis were also excluded; reference lists of relevant reviews identified by the search strategy were hand-searched for additional eligible studies.

### Data extraction

The titles and abstracts of identified papers in the main search were screened independently by three reviewers, applying the inclusion and exclusion criteria. Full text of retained references were then obtained and screened independently by two reviewers, using the same inclusion and exclusion criteria. Disagreements were resolved by discussion.

Results from the 2021 update search were initially screened by one author, and abstracts and full-text papers were then reviewed by two reviewers to determine studies to be included for full review.

Data extraction was carried out by two reviewers using Microsoft Excel. Two forms were developed, one for importing general information from the selected studies: names of the authors, date of publication, the research country, resilience definition, purpose of resilience (what goals and objectives it was supporting), focus of resilience (what triggered resilience, for example shock or type of stressor), what resources were involved (what components, resources or participants were involved), and the processes through which the healthcare organisations were able to be resilient (the mechanisms, activities and interactions that supported resilience). Another form was used to extract information on resilience assessment or measurement, and included: format (tool, model, checklist, survey, interview, modelling, etc.); methods (e.g., quantitative, qualitative, mixed); what was measured or assessed (capacities, outcomes); when (pre-, during or post-shock), and any other relevant information relating to assessing or measuring resilience in healthcare organisations. The process was repeated for the studies from the updated searches in 2021.

### Quality assessment of studies

We assessed the quality of the included studies using simple criteria based on appropriate and published checklists [34]. We assessed the studies on their methodologies and their interpretations of findings.

### Synthesis and reporting

We initially categorised studies based on their focus on either measurement (quantitatively measuring levels/presence/absence of resilience) or assessment of resilience (qualitatively assessing resilience, identifying how resilience is created, maintained or broken down) [35]. We then conducted a descriptive analysis of the included studies. Data were tabulated to compare the approaches, including the assessment or measurement goals, data collection methods and the constructs/dimensions assessed or measured. This process of comparison across studies led us to identify thematic areas of contrast across approaches, with emphasis on their relevance to informing decisions by researcher, healthcare managers, and policymakers about selecting appropriate measures or assessment approaches. Data synthesis was conducted by a single reviewer and validated by two other reviewers. All authors of this manuscript contributed to refining the results of the review.

## Results

### Description of included studies

**Fig. 1:**
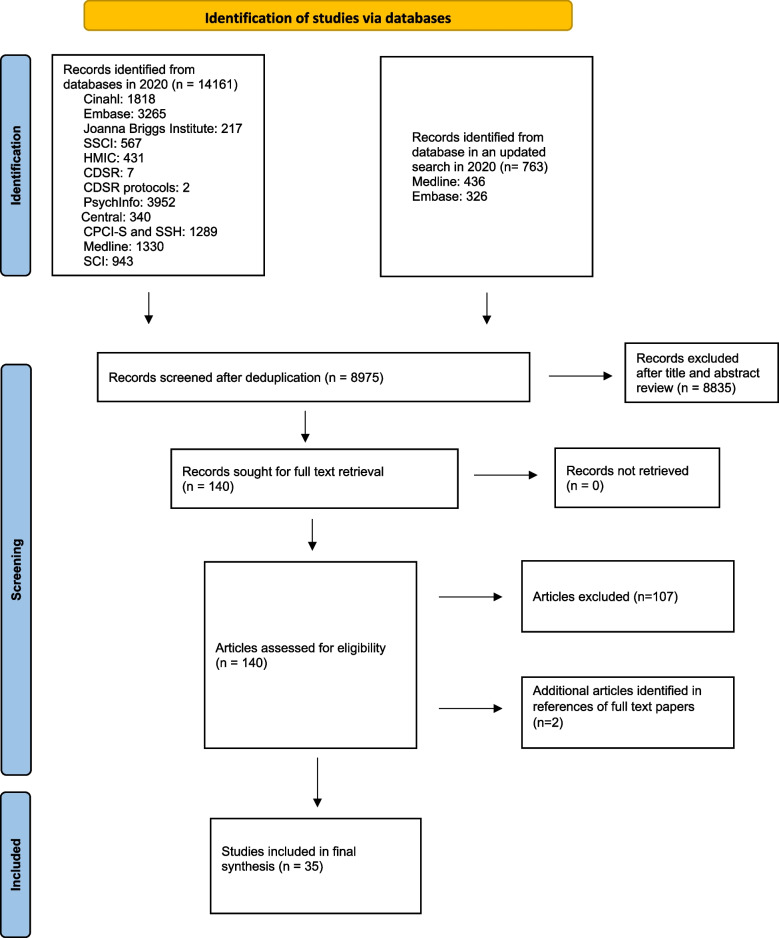
PRISMA flow diagram of study selection process

The main searches yielded a large number of references (14,161). After systematic screening we included 30 of those articles in the full review. We identified two additional articles from reference mining. The updated searches in 2021 identified three additional articles. In total, 35 studies that measured or assessed organisational resilience in the context of healthcare were included in the final synthesis (Fig. 1).

All studies focused on measuring or assessing resilience of hospitals or healthcare facilities. Thirty-one studies involved quantitative measurement; four studies involved qualitative assessment. The largest number of studies were undertaken in the United States (n = 7) and Iran (n = 6), with the remainder being conducted in a range of high and low to middle income countries. Two studies compared resilience in different countries (South Africa and Kenya, n = 1 and Iran and Sweden, n = 1). Most studies focused on resilience to acute shocks (n = 31): 22 of these focused on specific events or shocks including natural disasters, mass casualty events, man-made conflicts and emerging infectious disease and pandemics; nine studied resilience in the context of unspecified disasters. The remaining four studies focused on chronic stressors/everyday resilience and studied resilience to changing demands and service reconfigurations. The table with the characteristics of the included studies is provided in Additional file 2.

### Approaches to organisational resilience measurement and assessment

We identified five thematic areas of contrast across the measurement and assessment approaches: (1) type of shock; (2) stage of resilience; (3) range of characteristics and indicators (4) nature of output; and (5) purpose (formative/diagnostic/summative—adapted from Mannion et al. [36]). Table 1 provides a summary of measurement and assessment approaches of organizational resilience included in this review, with the characteristics and indictors used in each study described in the appendix.

**Table 1:** Measurement and assessment approaches of organizational resilience

Author/s, year	Format of the approach	Type of shock/challenge	Stage of resilience	Nature of output	Purpose
Adini et al. 2012 [37]	Structured evaluation tool	Various types of emergencies (mass casualty events, toxicological and chemical events, and biological events such as pandemics and bio-terror agents)	Pre-shock	Overall preparedness for emergencies score for each hospital	Formative, diagnostic
Awad and Cocchio, 2015 [38]	An electronic cross-sectional survey	Mass casualty events	Pre-shock	Percentages and frequencies describing preparedness level of hospital pharmacy for each survey question	Formative
Bin Shalhoub et al. 2017 [39]	Questionnaire based on the WHO toolkit for assessing health-system capacity for crisis management and WHO hospital emergency response checklist	Mass casualty events	Pre-shock	Percentages and frequencies describing preparedness level of hospitals for each question or area of the questionnaire	Formative, diagnostic
Higgins et al. 2004 [40]	A survey instrument based on the Mass Casualty Disaster Plan Checklist and a brief supplemental bioterrorism preparedness survey based on a checklist developed for the Agency for Healthcare Research and Quality	Mass casualty events	Pre-shock	Percentages and frequencies describing preparedness level of hospitals	Formative, diagnostic
Traub et al. 2007 [41]	Hospital surge capacity survey	Mass casualties as a result of terrorism or natural disasters	Pre-shock	Numbers of operating theatres, ICU beds and x-ray machines required according to the predictor of numbers of mass casualties	Formative, summative
Aladhrai et al. 2015 [42]	WHO Hospital emergency response checklist	Man-made conflicts (2011 Yemeni revolution)	Pre- and post-shock (comparing the level of preparedness in 2011 with that in 2013)	Overall preparedness score for each hospital Hospitals categorised as unacceptable, insufficient or effective, based on the overall score	Formative, diagnostic
Ardalan et al. 2016 [43]	Farsi version of the WHO Hospital Safety Index (FHSI)	Disasters	Pre-shock	Overall score of hospital safety Hospitals categorised to three safety classes: low, average, and high based on the total score	Formative, diagnostic
Djalali et al. 2013 [44]	The Functional Capacity module of the WHO Hospital Safety Index (HSI)	Disasters	Pre- and during the shock	Functional capacity score for each hospital Hospitals categorised as: (1) will function during a disaster; (2) at risk, interventional measures are needed; (3) inadequate, urgent intervention is needed	Formative, diagnostic
Zhong et al. 2014 [45]	A framework and derived questionnaire for measuring hospital disaster resilience	Disasters	Pre-, during and after	Overall hospital resilience score, hospitals classified as extremely resilient to disasters; extremely impacted upon in a disaster; or have greater difficulty in recovering based on the overall score	Formative, diagnostic
Janati et al. 2017 [46]	WHO hospital emergency response checklist	Disasters	Pre-shock	Percentages and frequencies describing preparedness level of hospitals	Formative, diagnostic
Naser et al. 2018 [47]	WO Hospital Emergency Response Checklist	Disasters	Pre-shock	Overall emergency preparedness and response score for each hospital Hospitals categorised as unacceptable, insufficient, or effective	Formative, diagnostic
Hosseini et al. 2019 [48]	Questionnaire based on the WHO Hospital Safety Index (HSI) and the assessment of vulnerability elements at hospitals developed by Mulyasari et al. (2013) Data analysed using TOPSIS technique	Unexpected disasters and events	Pre-shock	Hospitals ranked based on disaster preparedness scores	Formative, diagnostic
Khazaei Monfared et al. 2017 [49]	WHO Hospital Safety Index translated to Farsi and adapted to Iran’s context by Ardalan et al. (2016)	Unexpected disasters (floods, earthquakes, severe weather changes, bioterrorism, and epidemics)	Pre-shock	Hospital safety score for each hospital Hospitals categorised as low safety, medium safety and high safety based on the safety score	Formative, diagnostic
Cimellaro et al. 2017 [50]	Discrete event simulation (DES) models	Disasters and other emergencies	During-shock	Model of an emergency department’s performance based on different parameters	Formative
Shirali et al. 2016 [51]	Questionnaire based on the seven dimensions of resilience engineering	Natural and man-made disasters	Pre-, during and post-shock	Average scores for each indicator related to the hospital in different crisis management phases	Formative, summative and diagnostic
Ul-Haq et al. 2019 [52]	WHO Toolkit for assessing health system capacity for crisis management (user manual and assessment form)	Natural or manmade, predictable or unpredictable disasters	Pre-shock	Percentages and frequencies describing preparedness level of hospitals for different indicators Healthcare facilities categorised as acceptable, partial or inadequate	Formative, diagnostic
Sobhani et al. 2014 [53]	A standard checklist	Natural disasters	Pre-shock	Overall level of preparedness against disasters for each hospital Hospitals categorised into very poor; poor; moderate; good; and very good	Formative, diagnostic
Brevard et al. 2008 [54]	Direct observation and recording of events from within the hospital before, during, and after the storm; retrospective review of the hospital master disaster plan and a survey of key staff present during and post-storm	Hurricanes	Pre-, during- and post-shock	Percentages and frequencies describing preparedness level; qualitative description of events from before, during, and after the storm; survey responses rated on a scale of three: completely inadequate, partially adequate, completely adequate	Formative, diagnostic and summative
Rios et al. 2021 [55]	Interviews with healthcare professionals Data analysed using Kruk et al.’s resilience framework	Hurricanes	Post-shock	Qualitative description of the key factors that led to a poorly resilient hospital and health system	Formative, summative
Cimellaro et al. 2010 [56]	A comprehensive model to quantify disaster resilience	Earthquakes	Pre-, during and post-shock	Loss estimation models and recovery models that can be applied to complex systems of structures and infrastructure networks	Formative
Jacques et al. 2014 [57]	Damage and loss-of-function survey tool Data analysed using fault-tree method	Earthquakes	Pre-, during and post-disaster	Descriptions of the loss of functionality of physical systems, the impact to healthcare services and support services, and the sharing of resources and transfer of patients in a hospital system; Variation in hospital functionality over time	Formative
Miniati and Iasio, 2012 [58]	The theory of complex systems analysis with the use of an input–output inoperability (Leontief) model and a rapid seismic vulnerability assessment with the field data collection using the WHO evaluation forms	Earthquakes	During-shock	Description of the hospital response evaluation with a focus on capacity to cope during an earthquake disaster based on different scenarios	Formative, diagnostic
Mulyasari et al. 2013 [59]	Questionnaire based on the WHO Hospital Safety Index (HSI) and the assessment of vulnerability elements at hospitals	Earthquakes	Pre-shock	Description of responses of the hospitals concerning the different preparedness indicators and past disaster experiences	Formative, diagnostic
Yavari et al. 2010 [60]	Predictive model for estimating the post-disaster ability to provide services	Earthquakes	Post-shock	A practical model for estimating the functionality of hospitals after an earthquake	Formative
Paterson et al. 2014 [61]	A climate change resiliency assessment toolkit (checklist, a facilitator’s guide for administering the checklist, and a resource guidebook to inform adaptation)	Climate change impact (extreme weather events)	Pre-shock	The degree to which healthcare facility is resilient to current climate variability and future climate change based upon included indicators	Formative, diagnostic
Ten Eyck, 2008 [62]	Standardized data form evaluating surge response plans The surge in demand was calculated using the FluAid and FluSurge tools	Hospital surge capacity as a result of avian flu pandemic	Pre- and during-shock	Cumulative surge capacity for the region Percentages and frequencies for each of the six categories of resources	Formative
Sharma and Sharma 2020 [63]	A semi-structured online questionnaire and available published and unpublished data for situation analysis	Pandemics (Covid-19 pandemic)	Pre-shock	Percentages and frequencies describing preparedness level of hospitals for each question or area of the questionnaire	Formative, diagnostic
Prateepko and Chongsuvivatwong, 2012 [64]	A checklist for health care facilities based on the WHO checklist for influenza pandemic preparedness planning, the preparedness checklist for long-term care facilities, other international infection control and preparedness checklists	Influenza pandemic	Pre-shock	Description of responses of the healthcare facilities concerning the different preparedness areas	Formative, diagnostic
Dewar et al. 2014 [65]	Questionnaire and semi-structured interviews	Pandemic influenza	Pre-shock	Percentages and frequencies describing preparedness level of hospitals and common themes emerging from interviews	Formative
Ambat and Vyas, 2020 [66]	Semi-structured questionnaire	Emerging infectious disease	Pre-shock	Percentages and frequencies describing preparedness level of hospitals	Formative
Gilson et al. 2017 [67]	Document reviews, in-depth interviews, group discussions and observations	Routine challenges	Pre-, during- and post-challenge	Qualitative description of different types of strategies and organizational capacities in nurturing everyday resilience	Formative, summative and diagnostic
Gilson et al. 2020 [68]	Observations, in-depth interviews, analysis of meeting minutes and secondary data Analysis of data based on the everyday health system resilience (EHSR) framework	Chronic stress of large-scale organizational change	Pre-, during- and post-challenge	Qualitative description of different types of strategies and organizational capacities in nurturing everyday resilience	Formative, summative and diagnostic
Kagwanja et al. 2020 [69]	Observations, reflective meetings and in-depth interviews with middle-level managers and peripheral facility managers Data were analysed considering each element of the everyday health system resilience (EHSR) framework	Chronic stressors	Pre-, during-and post-challenge	Qualitative description of different types of absorptive, adaptive and transformative strategies and organizational capacities in nurturing everyday resilience adopted	Formative, summative and diagnostic
Crowe et al. 2014 [70]	A model and prototype software tool	Disruptions due to service reconfigurations	During-and after challenge	Model of the impact of different patterns of disruption to healthcare resources and infrastructure on health services	Formative
Davis et al. 2020 [71]	Modelling framework	Hospital overcrowding	During-shock	Model quantifying the impact of each contributing component on hospitals	Formative

### Type of shock

Identified measurement and assessment approaches focused on three qualitatively different types of shock or challenge: (1) resilience to disasters (including unexpected and natural disasters, unspecified disasters, and disastrous events causing mass casualties); (2) resilience to infectious diseases (including influenza, emerging infectious disease and avian flu pandemics); and (3) resilience to chronic challenges and fluctuating levels of demand for healthcare (everyday challenges, disruptions due to service reconfigurations and organizational change). These domains differed in the likelihood and predictability of the shock or challenge, the timeline over which the shock or challenge unfolded, and the nature of the ‘post-shock’, or recovery, period.

Across the three types of shock or challenge, different formats were used to evaluate organizational resilience. The most common formats included surveys and questionnaires (n = 9), checklists (n = 5), tools/toolkits (n = 4) and quantitative modelling (n = 6). Within the resilience to disasters and domain, some approaches utilised already existing and validated instruments, in original or adapted form, such as the World Health Organisation (WHO) Hospital Safety Index (HSI) [72], the WHO Hospital Emergency Response Checklist [73], or the WHO toolkit for assessing health-system capacity for crisis management [74]. In contrast, approaches focusing on resilience to infectious diseases and chronic stressors/fluctuating levels of demand more commonly developed new instruments.

### Stage of resilience

A significant proportion of studies, particularly those that evaluated resilience in the context of organizational disaster preparedness and emergency management response, included measurement and modelling of the state of preparedness and plans for responding to an acute event. These approaches focused on capacity to operationalise resources in the event of a shock or challenge, and as such aimed to measure resilience prospectively, in a pre-shock stage. For instance, Ambat and Vyas [66] surveyed different staff members in five hospitals in South India to evaluate the level of hospital preparedness against emerging infectious disease. The modelling approaches attempted to predict future stressors and their consequences. Davis et al. [71] presented a predictive model based on the different components that combine the US National Emergency Department Overcrowding Scale (NEDOCS) score to determine when overcrowding is likely to occur, and tested it on the data from past disaster-level overcrowding events in the emergency departments.

The remainder of the studies included other or a combination of stages of resilience. Brevard et al. [54] evaluated a major trauma centre’s response to hurricane by reviewing the hospital master disaster plan retrospectively and comparing it with the actual events that occurred. They also undertook a survey of emergency medicine and surgery residents, and staff present during the hurricane and through the evacuation period. Miniati and Iasio [58]  modelled hospital’s response to earthquakes based on the damage to structural, nonstructural and organisational factors, whereas Yavari et al. [60] used available data from previous earthquakes to develop a predictive model for estimating hospital’s post-disaster ability to provide services. Studies that used a qualitative assessment approach tended to consider multiple stages of resilience. Gilson et al. [68], for instance, tested the everyday health system resilience (EHSR) [16] framework and examined how health managers and staff in one local health system in South Africa manged and responded to the chronic stress of large-scale organisational change. Kagwanja et al. [69] investigated strategies and organisational capacities adopted by middle-level (sub-county and hospital) managers and frontline peripheral facility managers facing the rapid devolution process and nationwide policy changes in Kenya. They then presented the strategies according to the EHSR framework, focusing on the underlying organisational capacities that enabled and blocked various responses to stressors in different stages of the resilience cycle.

### Characteristics or indicators included

The characteristics or indicators used for measuring and assessing organizational resilience varied across the approaches. Some of the approaches used very narrow list of characteristics, while other had a much more extensive list for consideration. The choice of characteristics or indicators was often based on understanding and framing of resilience, and in some cases informed by reviews of literature, reports, and international guidelines. Although most approaches used pre-defined sets of characteristic or indicators, some (mainly qualitative assessment approaches) explored the nature and extent to which any of the characteristics were present in an organization.

For example, in the context of measuring resilience to disasters, Shirali et al. [51] evaluated resilience based on seven dimensions of resilience engineering: top management commitment, just culture, learning culture, opacity, preparedness, awareness and flexibility, whereas Zhong et al. [45] proposed characteristics based on resilient theory: hospital safety, emergency services, surge capacity, command, disaster plan, logistics, staff ability, disaster training, communication and cooperation systems, recovery, and adaptation. In the context of measuring resilience to infectious diseases, Ambat and Navya [66] organised their survey questions according to various domains of the International Health Regulations (IHR) framework. The qualitative assessment approaches focusing on chronic challenges and fluctuating levels of demand for healthcare tended to use frameworks. For instance, Kagwanja et al. [69] evaluated strategies and organisational capacities adopted by clinical managers in the study against the constructs in the EHSR framework to understand the processes underpinning resilience, and the different strategies and organisational capacities that fostered it.

The types of characteristics or indicators varied widely, with different approaches proposing particular ways of grouping indicators based on the purpose of the evaluation and the shock studied. Approaches within the resilience to disasters domain tended to examine hospital or healthcare facility characteristics that predicted disaster preparedness, and often used operational characteristics of healthcare organizations as their indicators. For instance, approaches utilising the WHO HIS measured hospitals' safety and vulnerabilities to undefined disasters based on structural (e.g., structural safety of the buildings), non-structural (equipment, resources, supplies) and emergency and disaster management (e.g., medical response; critical systems, hospital emergency and disaster management response and recovery planning) indicators. Some approaches within this theme also focused on preparedness in terms of the capacity to respond to the disasters and included infrastructure, resources and post-disaster plans as their indicators (e.g., Shirali et al. [51] and Awad and Cocchio [38] in the context of disasters associated with mass casualties). Measurement and assessment approaches exploring resilience to infectious diseases also tended to examine a range of hospital and healthcare facility characteristics, with a broad focus on operational characteristics but also capacity to deal with infectious diseases. For example, Prateepko and Chongsuvivatwong [64] grouped their indicators under the 5 main areas: facility access plan, epidemiological surveillance, infection control, risk communication and health information dissemination, and health alert network and information technology, whereas Dewar et al. [65] covered hospital planning information, workforce issues, infrastructure and surge capacity in their questionnaire. Approaches that employed modelling and scenario analysis were used to predict unmet demands and their consequences and tended to use indicators associated with the capacity to continue the delivery of care (surge capacity, patient waiting times, the number of beds in use and staffing schedules) and in relation to specific shocks (i.e., earthquakes). The qualitative assessment approaches covering resilience to chronic challenges and fluctuating levels of demand for healthcare mainly relied on the characteristics and indicators aligned with the theoretical frameworks used in these studies and covered a wide range of strategies and organizational capacities that were associated with resilience.

There were also significant differences between the approaches in terms of the number of characteristics and indicators included, for example, Adini et al. [37] measured resilience to mass causality disasters based on 490 indicators, whereas Rios et al. [55] assessed the everyday resilience on the basis of five foundational components of Kruk’s resilience framework [75]. The established WHO diagnostic instruments focusing on undefined disasters had multiple components with sub-questions, pushing the number of indicators to 145 or more.

### Nature of output

The reviewed approaches expressed organizational resilience in one of four ways: (i) overall score of resilience and/or classification of an organization into different categories based on that score; (ii) presence or absence of resilience characteristics and indicators, usually described in frequencies or percentages for all areas of interest; (iii) quantitative model/models to predict how the organization may respond based on different factors; or (iv) qualitative description of healthcare organisation’s responses to challenges. For instance, Adini et al. [37] expressed resilience as an overall score of readiness for emergencies; Sharma and Sharma [63] used percentages and frequencies to describe the preparedness level; and Cimellaro et al. [56] modelled resilient performance based on different parameters.

### Purpose

Considering their practical application, we classified the approaches into three groups based on their intended purpose: formative, summative and diagnostic (adapted from Mannion et al. [36]) – some approaches could fulfil more than one purpose. Formative approaches can provide ongoing feedback and can be used to inform organizational development and learning with regards to resilience. Summative approaches can provide an evaluation of achievement, or failure, in respect of intended resilience goals and interventions. Diagnostic approaches can be used to provide information on strengths and weakness in an organisation and ascertain, prior to any intervention, where the organisation is in respect to their resilience goals. All these approaches aim to provide information on resilience, but with different focus and outcomes.

The WHO toolkit for assessing health-system capacity for crisis management [74], Traub et al.’s [41] hospital surge capacity survey and Adini et al.’s [37]  structured evaluation tool are examples of formative approaches, measuring the capacity of organizations to respond to different disasters. These approaches are self-reported in nature, usually completed by the evaluation team, staff in an organization or the hospital/healthcare facility management. They can be used to monitor progress with regards to resilience, for example, by measuring improvement in preparedness to shocks over time, and to aid learning with regards to development of the organization.

A number of approaches can be used as summative approaches. For example, Shirali et al.’s [51] crisis management questionnaire, based on the seven dimensions of resilience engineering, measures resilience to natural and man-made disasters in four phases, i.e., prevention, preparedness, response, and recovery (i.e., pre, during-and post-shock). Results from the application of this summative approach can be used by hospital managers, emergency planners and policymakers to help measure the capacity of the hospital to cope with a range of potential shocks across the various stages of the crisis management cycle. Similarly, Zhong et al.’s [45] framework and questionnaire measures preparedness before disasters but also includes capacity to deal with recovery and adaptation after the disaster. The derived resilience scores can be used to evaluate key indicators of hospital resilience.

These, and other summative approaches can help frame policy discussions regarding the need to strengthen resilience, as well as identify those hospitals and healthcare facilities that seem to be especially vulnerable. This information can provide input into funding and prioritization decisions.

Finally, diagnostic approaches can facilitate an understanding of the weaknesses and strengths in relation to resilience and help develop plans and interventions to address these. The WHO HSI [72] is an example of a diagnostic approach—it has been designed to evaluate the probability that a hospital will remain operational in case of a disaster. The Index yields useful information about a hospital’s strengths and weaknesses and points to the actions required to improve the safety and emergency and disaster management-capacities. Similarly, Paterson et al.’s [61] climate change resiliency assessment toolkit helps healthcare facility officials identify gaps in climate change preparedness, direct allocation of adaptation resources and inform strategic planning to increase resiliency to climate change. Therefore, specific interventions can be developed to address the areas that are weak or that lack the capacity. The diagnostic approaches can highlight the basic benchmarks for further works to cover the areas that were not reached by the intervention. When completed, the toolkit can also provide information that can feed into regular planning and ongoing decision-making. It can therefore be used as a formative approach to guide organizational development and learning.

## Discussion

This systematic review has deliberately sought to identify approaches to assessment and measurement of organisational resilience in healthcare. Our search identified 35 papers that reported on approaches to measurement and qualitative assessment of organisational resilience in the healthcare context, which had been piloted or tested in practice. We identified five domains across which the measures varied: the type of shock, the stage(s) of resilience being assessed, the included characteristics and indicators, the nature of the output, and whether the approach was intended to be used formatively, as a diagnostic tool, or summatively. This review provides insight into the diversity of tools and approaches for measuring organisational resilience in healthcare and invites some reflections on the field.

First, there is no consensus on how to evaluate resilience, what should be measured or assessed, when, and using what indicators. The different approaches reviewed here offer different insights into organizational resilience. As others have noted, different perspectives place different emphasis on the resilience concept, and this influences how resilience is evaluated, what data is collected and using what indicators [76, 77]. Our review demonstrates how differences in the focus and the aims underpinning the development of different resilience measures have resulted in a proliferation of diverse tools and approaches.

Approaches to resilience measurement and assessment varied in scope, format and purpose. The approaches varied in terms of whether they were measuring or assessing resilience prospectively (pre-shock), retrospectively (post-shock) or covered more than one resilience stage, and the extent to which they addressed a pre-defined and shock-specific set of characteristics and indicators. For example, the approaches in the disaster domain tended to focus on resilience in the preparedness stage, with some approaches extending their evaluation to other stages. They also measured or assessed resilience using either a small and narrow selection of resilience characteristics (e.g., functionality of a hospital) or a set of more comprehensive characteristics (e.g., structural, non-structural, functional safety of a hospital). The resilience characteristics were shock-specific (or grouped based on similar shocks) and pre-defined. The approaches in the infectious disease thematic domain were also characterised by dominant interest in the pre-shock stage, focusing on operational characteristics of the organizations and their capacity to deal with potential infectious diseases, but also considered the organisational response to the shock. Modelling approaches focusing on chronic stressors/fluctuating levels of demand for healthcare tended to quantify resilience on a basis of functionality and measured the impact of the challenge on the organization. They described management capabilities that organisations developed in each resilience stage through coping with unexpected events and everyday challenges. The qualitative approaches in this domain used explicit resilience frameworks; these frameworks were used to indicate the information needed to be collected to evaluate resilience and/or guided data analysis. These qualitative approaches also assessed organisational resilience during and post-challenge, and generally focused on a broader set of resilience characteristics.

Given this diversity of available approaches, there are issues to be considered when considering the practical application of the approaches. Because of the characteristics of quantitative measurement approaches, it can be argued that these are more appropriate for performance benchmarking or measuring resilience status or level (formative and diagnostic purposes). These approaches can also help decision-makers identify areas that need to be strengthened and prioritized (diagnostic purposes) or utilized to monitor effectiveness and efficiency of their plans and policies (formative purposes). Modelling approaches, in particular modelling capacity to recover or developing different impact scenarios based on potentially relevant variables, can help aid organizational learning and planning. Qualitative assessment approaches are more flexible and can be more easily adapted. Since they provide opportunities to understand what resilient organisations do and how organisational resilience may be achieved in practice, they may be best used to consider as strategies for building and enhancing resilience (formative and diagnostic purposes). These approaches may also be most valuable for learning about the direct management of shocks and challenges and further areas for resilience evaluation.

### Recommendations

A range of approaches with differing characteristics and indicators are available to researchers, policymakers and healthcare managers interested in evaluating organizational resilience. Given the current lack of consensus and diversity in available tools and approaches for measuring and assessing organisational resilience in healthcare, we describe the key issues that should be considered when attempting to choose between the evaluation approaches:

1. The choice of an approach should be determined by the type of shock, the purpose of the evaluation, intended use of results, and availability of data and resources. Other factors to consider are timelines for evaluation, and target audience and the needs of different stakeholder (Sturgess [11]; Quinlan et al. [35]; Schipper and Langston [78]). The way resilience is framed impacts on the type of evaluation approach that is considered most effective in any given context. Researchers, policymakers and healthcare managers need to ensure the compatibility between the approach, the aims of the evaluation and resources available.
2. The characteristics and indicators of resilience included in measurement or assessment approaches should reflect the type of the shock or challenge, and the purpose of the evaluation. The literature on resilience suggests that there are likely to be inherent trade-offs involved in measuring a narrow set of indicators (Quinlan et al. [35]), but also challenges with defining the characteristics that lead to resilience prior to undertaking measurement as the conclusions will be largely driven by the initial selection of these characteristics (Cumming et al. [79]). It is also difficult to design and choose generalizable indicators for qualitatively assessing resilience because these approaches tend to focus on understanding how resilience works. Researchers, policymakers and healthcare managers need to be clear about what they are measuring or assessing, and why. They may also need to decide which characteristic and indicators need to be prioritised.
3. Different methodologies and formats may be needed, depending on the purpose of evaluation. Researchers, policymakers and healthcare managers may need to decide what type of approach is most beneficial, under what conditions, and in which organisations. Some approaches can be used for more than one evaluation purpose and there may be trade-offs in how best to design approaches so that they can serve more than one purpose. A mix of quantitative and qualitative approaches may be needed to produce a more useful insight into resilience. There is a growing recognition that resilience should be understood as both an outcome and a process (EU Expert Group on Health System Performance Assessment [3]; Duchek [4]; Winderl [76]). Researchers, policymakers and healthcare managers may also wish to consider adopting a multimethod and ongoing approach to evaluation (Quinlan et al. [35]). Quantitative measurement can play a role in regular monitoring and reporting prior to a shock or comparing levels of resilience prior and after the shock or challenge, whilst qualitative assessment can help evaluate the impact of resilience interventions, identify opportunities and different strategies to inform decision-making during and post-shock. Modelling and projection approaches can be used to account for different scenarios and minimise the influence of uncertainties on the decision-making process. When using a mix of approaches, the need for researchers, policymakers and healthcare managers to consider how resilience, and the characteristics and indicators that contribute to it, change over time is essential. This may mean tailoring approaches to different needs and end-users and considering the influence that data from one approach may have on another. Understanding how different approaches and formats to evaluation can address resilience dynamics over time has been highlighted as an area for further work in the literature (Quinlan et al. [35]; Biddle et al. [8]).
4. Finally, it should be noted that resilience assessment and measurement is still a developing field. Besides the conceptual challenges, there are also methodological and practical challenges such as data availability and accuracy, development of indicators, choice of aggregation or weighting, etc. that should be considered (for an overview of conceptual and methodological challenges around measurement and assessment of resilience, see Levine [77] and Quinlan et al. [35]). The approaches reviewed here have been designed and used in specific contexts and often for the purposes of the study. Researchers, policymakers and healthcare managers will most likely have to adapt the approach and add or remove characteristics and indicators according to their specific needs and priorities. More empirical research is needed to investigate the applicability and utility of assessment and measurement approaches to provide information on possible strategies for better integration of resilience evaluation for decision-making and planning. Further work is also needed to elaborate on how the qualitative and quantitative approaches can complement each other (Quinlan et al. [35]; Biddle et al. [8]).

Overall, our results provide a state of the art summary of the range of approaches used in empirical studies to measure organisational resilience in healthcare, to inform healthcare researchers, policymakers and managers interested in evaluating resilience at the organizational level.

The diversity of measurement approaches means that data from different studies lack comparability, undermining any efforts to synthesise evidence about resilience across studies. Ongoing conceptual work has generated integrative theoretical models that could underpin the development of future approaches to the measurement and assessment of resilience [14, 80]. However, the shift from conceptual research, to research with practical implications requires recognising the dynamics and complexities of resilience [22]. This review notes that organizational resilience is likely to be influenced by the dynamic relationships and interdependencies that may exist beyond the meso level, and very few empirical studies have addressed the inherent complexities of resilience within and across system levels [81]. Consequently, there is a need for empirical studies that describe resilience at multiple levels of healthcare system, from individual healthcare staff (micro), to organisations (meso), and regulators and policymakers (macro), with approaches to assessment and measurement flexible enough to accommodate the dynamic nature of resilience [22, 82].

Further work to develop theory-informed methods for measuring and assessing organisational resilience in healthcare is urgently needed. A shared understanding of resilience would have value in addressing some of the challenges in the resilience measurement and assessment filed. A considerable amount of progress has been made on understanding the concept [16, 19, 80]. However, as this review indicates, the benefits gained through a shared understanding of the concept must be appropriately tuned to a particular context. An understanding of the context in which resilience is being evaluated is needed to determine the appropriate approach to assessment and measurement [35].

### Review strengths and limitations

The review included a focused but comprehensive overview of available tools and approaches for assessment and measuring resilience. Most of the included studies were quantitative. The quality of reporting was typically good: the reporting of results and discussion sections was generally strong with most providing indications of key findings and limitations. Weaknesses were identified in some publications in the reporting of study design, methods, and interpretation of data. Although all the measures and approaches included in the review had been piloted or tested, few had been subject to rigorous validation or reliability testing (except for those that used the WHO tools). The review methods had some limitations. Firstly, in order to retrieve a manageable number of records with a high chance of relevance, search terms focused on organizational resilience in the healthcare context. We therefore might have missed empirical studies that used different terminology or studies in associated disciplines with relevance to healthcare. We also did not include non-English publications due to time constraints, which may have provided further useful information. However, the use of MeSH terms, broad inclusion criteria and hand-searching for additional eligible will have helped to retrieve some relevant records that would otherwise have been missed. Secondly, the review was limited to studies published up to September 2021, hence we have not identified studies of resilience published post-pandemic. Thirdly, this review identified a significant bias towards research into disaster preparedness, which could have limited the applicability and usefulness of findings. Finally, this review was primarily descriptive, aiming to summarise the existing approaches to organizational resilience measurement and assessment. Due to the diversity of the characteristics and indicators included in the measures, we did not aim to generate a comprehensive synthesis of indictors of organisational resilience included across measures and approaches. The wide variation in the number and nature of indicators and characteristics is likely to reflect both the theory or model of resilience that informed to development of the approach, as well as the specific context and aims of the approach.

## Conclusions

This review provides an overview of approaches to evaluation of organizational resilience in the healthcare context. The included studies used diverse approaches to quantitative measurement and qualitative assessment of resilience, with different conceptualizations, formats and methods of data collection. An important finding of this review is that there is currently no consensus on how to evaluate organisational resilience in healthcare, what should be measured or assessed and using what characteristic and indicators. The measurement and assessment approaches varied in scope, format and purpose. Some focused on the evaluation of resilience at one stage of the resilience process and addressed only particular aspects of resilience, others took a much broader perspective, with resilience evaluation undertaken at multiple stages and using a variety of indicators. When considering the practical applications of these different approaches for research and policy, this review argued that the choice of an approach should be determined by the type of shock, the purpose of the evaluation, intended use of results, availability of data and resources.

## Supplementary Information

**Additional file 1.** Example of search strategies for models of resilience topic: All searches run early Sept 2020.

**Additional file 2: Appendix.** Characteristics of included studies.

## Data Availability

All data generated or analysed during this study are included in this published article and its supplementary information files.

## Abbreviations

NIHR: National Institute for Health Research
PRP: Policy Research Programme
PRISMA: Preferred Reporting Items for Systematic Reviews and Meta-Analysis
WHO: World Health Organisation
WHI HIS: World Health Organisation Hospital Safety Index
NEDOCS: National Emergency Department Overcrowding Scale
EHSR framework: Everyday health system resilience framework
IHR framework: International Health Regulations framework
