# Correction: Organizational resilience in healthcare: a review and descriptive narrative synthesis of approaches to resilience measurement and assessment in empirical studies

**DOI:** 10.1186/s12913-023-09636-9

**Published:** 2023-07-05

**Authors:** Agnieszka Ignatowicz, Carolyn Tarrant, Russell Mannion, Dena El‑Sawy, Simon Conroy, Daniel Lasserson

**Affiliations:** 1 Institute of Applied Health Research, College of Medical and Dental Sciences, University of Birmingham, Birmingham, UK; 2 Department of Health Sciences, University of Leicester, Leicester, UK; 3 Russell Mannion, Health Services and Management Centre, College of Social Sciences, University of Birmingham, Birmingham, UK; 4 MRC Unit for Lifelong Health and Ageing, University College London, London, UK; 5 Warwick Medical School, University of Warwick, Coventry, UK

**Correction to**: *BMC Health Services Research* (2023) 23:376

10.1186/s12913-023-09242-9

Following publication of the original article [1], the authors reported an missing information in the ‘Funding’ section.

The statement in the ‘Funding’ section originally read: This study was funded by National Institute for Health Research (NIHR) Policy Research Programme (PRP) (Award ID: NIHR200718).

The statement in the ‘Funding’ section should read: This study was funded by National Institute for Health Research (NIHR) Policy Research Programme (PRP) (Award ID: NIHR200718). This is study was supported by the NIHR Applied Research Collaboration (ARC) WestMidlands, NIHR Community Healthcare MedTech and IVD Cooperative (MIC) and NIHR Oxford Biomedical Research Centre (BRC) through funding to DL.

The original article [1] has been updated.
