# Supplementary material for: Organizational resilience in healthcare: a review and descriptive narrative synthesis of approaches to resilience measurement and assessment in empirical studies
Source: BMC Health Serv Res. 2023 Apr 19;23:376. doi: 10.1186/s12913-023-09242-9 (PMC10113996; doi:10.1186/s12913-023-09242-9)
Supplement: Supplementary file 1 — Additional file 1. Example of search strategies for models of resilience topic: All searches run early Sept 2020. [file 12913_2023_9242_MOESM1_ESM.docx]

**Search strategies for models of resilience topic: All searches run early Sept 2020**

**Ovid MEDLINE(R) and In-Process & Other Non-Indexed Citations <1946 to Sept 10, 2020>**

1 exp Health Plan Implementation/ (5983)

2 (Plan adj8 resilien*).ti,ab. (40)

3 exp Program Development/ (29050)

4 (Program adj8 resilien*).ti,ab. (38)

5 (Protocol adj8 resilien*).ti,ab. (51)

6 (Paradigm adj8 resilien*).ti,ab. (83)

7 Checklist/ (6415)

8 Checklist.ti,ab. (34299)

9 (Tool* adj8 resilien*).ti,ab. (270)

10 (Framework adj8 resilien*).ti,ab. (512)

11 (Index adj8 resilien*).ti,ab.(159)

12 (Algorith* adj8 resilien*).ti,ab. (74)

13 (Indices adj8 resilien*).ti,ab. (63)

14 (Model* adj8 resilien*).ti,ab. (1449)

15 (Schem* adj8 resilien*).ti,ab. (55)

16 Safety II.ti,ab. (58)

17 Moments of resilience model.ti,ab. (1)

18 Integrated resilience framework.ti,ab. (0)

19 Concepts for applying resilience.ti,ab. (1)

20 WHO's six building blocks of a health system.ti,ab. (1)

21 The Hospital Safety Index.ti,ab. (8)

22 a capability-based organizational resilience framework.ti,ab. (0)

23 or/16-22 (69)

24 or/1-15 (73883)

25 23 or 24 (73944)

26 User involvement.ti,ab. (740)

27 IT solution*.ti,ab. (177)

28 (Robotic adj2 surgery).ti,ab. (5707)

29 Telecare.ti,ab.

30 (((DMAT or Disaster Medical Assist Team or DPAT or Disaster psychiatric assistance team or EMIS or Emergency Medical Information System or SPPED or Surveillance in Post-Extreme Emergencies) and Disasters) or Disaster Medicine* or Emergenc* Responder*).ti,ab. (997)

31 mHealth.ti,ab. (2962)

32 (Knowledge adj3 shar*).ti,ab. (3248)

33 (Bed adj3 manag*).ti,ab. (526)

34 ((disaster adj (drill or plan or prepare* or manage)) or (emergenc* adj (drill or plan or prepare* or manage))).ti,ab. (3797)

35 (capacit* adj3 plan*).ti,ab. (2502)

36 (Discharge adj3 plan*).ti,ab. (4426)

37 Emergency shelter.ti,ab. (101)

38 ((Robust* adj5 health) or (Strengthen* adj5 health)).ti,ab. (7652)

39 (Learn* adj5 health*).ti,ab. (10360)

40 Reorganis*.ti,ab. (2858)

41 (Flexib* adj5 health*).ti,ab. (1258)

42 ((Capabl* adj5 health*) or (Capabil* adj5 health*)).ti,ab. (2513)

43 (Resourceful* or (adapt* adj5 health)).ti,ab. (6134)

44 (Transform* adj5 health*).ti,ab. (4753)

45 (Recover* adj5 health*).ti,ab. (4883)

46 Resilien*.ti,ab. (31068)

47 exp Disaster planning/ (14279)

48 (Innovat* adj5 health*).ti,ab. (7142)

49 Health system*.ti,ab. (50679)

50 Health Institution.ti,ab. (754)

51 Health governance.ti,ab. (401)

52 Health organisation.ti,ab. (3699)

53 Global Health.ti,ab. (19480)

54 Delivery of Health Care/ (90923)

55 *Global Health/ (19608)

56 *Health Policy/ (35547)

57 (High income adj5 health*).ti,ab. (568)

58 (NHS or National Health Service).ti,ab. (39799)

59 Hospital*.ti,ab. (1259561)

60 health facilities/ or health services/ (39769)

61 (health service* or health facilit*).ti,ab. (118321)

62 healthcare setting.ti,ab. (2205)

63 or/38-48 (89814)

64 or/49-62 (1564571)

65 or/26-37 (25639)

66 63 or 65 (112366)

67 66 and 64 and 25 (1212)

**Database: Embase 1974 to 2020 September 10**

1 exp Health Care Planning/ (97940)

2 (Plan adj8 resilien*).ti,ab. (50)

3 exp Program Development/ (24159)

4 (Program adj8 resilien*).ti,ab. (569)

5 (Protocol adj8 resilien*).ti,ab. (60)

6 (Paradigm adj8 resilien*).ti,ab. (125)

7 Checklist/ (25026)

8 Checklist.ti,ab. (48011)

9 (Tool* adj8 resilien*).ti,ab. (374)

10 (Framework adj8 resilien*).ti,ab. (584)

11 (Index adj8 resilien*).ti,ab. (224)

12 (Algorith* adj8 resilien*).ti,ab. (82

13 (Indices adj8 resilien*).ti,ab. (74)

14 (Model* adj8 resilien*).ti,ab. (1775)

5 (Schem* adj8 resilien*).ti,ab. (55)

16 Safety II.ti,ab. (91)

17 Moments of resilience model.ti,ab. (1)

18 Integrated resilience framework.ti,ab. (0)

19 Concepts for applying resilience.ti,ab. (2)

20 WHO's six building blocks of a health system.ti,ab. (1)

21 The Hospital Safety Index.ti,ab. (12)

22 a capability-based organizational resilience framework.ti,ab. (0)

23 or/16-22 (107)

24 or/1-15 (177681)

25 23 or 24 (177775)

26 User involvement.ti,ab. (939)

27 IT solution*.ti,ab. (325)

28 (Robotic adj2 surgery).ti,ab. (10492)

29 Telecare.ti,ab. (774)

30 (((DMAT or Disaster Medical Assist Team or DPAT or Disaster psychiatric assistance team or IS or Emergency Medical Information System or SPPED or Surveillance in Post-Extreme Emergencies))) and Disasters) or Disaster Medicine* or Emergenc* Responder*).ti,ab. (1298)

31 mHealth.ti,ab. (3085)

32 (Knowledge adj3 shar*).ti,ab. (4609)

33 (Bed adj3 manag*).ti,ab. (774)

34 ((disaster adj (drill or plan or prepare* or manage)) or (emergenc* adj (drill or plan or prepare* or manage))).ti,ab. (4519)

35 (capacit* adj3 plan*).ti,ab. (3027)

36 (Discharge adj3 plan*).ti,ab. (6731)

37 Emergency shelter.ti,ab. (112)

38 ((Robust* adj5 health) or (Strengthen* adj5 health)).ti,ab. (9004)

39 (Learn* adj5 health*).ti,ab. (13456)

40 Reorganis*.ti,ab. (3945)

41 (Flexib* adj5 health*).ti,ab. (1558)

42 ((Capabl* adj5 health*) or (Capabil* adj5 health*)).ti,ab. (3189)

43 (Resourceful* or (adapt* adj5 health)).ti,ab. (7766)

44 (Transform* adj5 health*).ti,ab. (5632)

45 (Recover* adj5 health*).ti,ab. (6620)

46 Resilien*.ti,ab. (38258)

47 exp Disaster planning/ (12526)

48 (Innovat* adj5 health*).ti,ab. (9390)

49 Health system*.ti,ab. (71083)

50 Health Institution.ti,ab. (1079)

51 Health governance.ti,ab. (429)

52 Health organisation.ti,ab. (5886)

53 Global Health.ti,ab. (26550)

54 Health Care delivery/ (174164)

55 *Global Health/ (3302)

56 *Health Policy/ (57457)

57 (High income adj5 health*).ti,ab. (677)

58 (NHS or National Health Service).ti,ab. (59677)

59 Hospital*.ti,ab. (1959056)

60 health facilities/ or health services/ (210834)

61 (health service* or health facilit*).ti,ab. (145058)

62 healthcare setting.ti,ab. (3310)

63 or/38-48 (107866)

64 or/49-62 (2479025)

65 or/26-37 (36420)

66 63 or 65 (141177)

67 66 and 64 and 25 (3746)

**Database: APA PsycInfo <1967 to September Week 2 2020>, APA PsycInfo <1806 to 1966>**

1 exp Health Care Delivery/ (98551)

2 (Plan adj8 resilien*).ti,ab. (42)

3 exp Program Development/ (9410)

4 (Program adj8 resilien*).ti,ab. (599)

5 (Protocol adj8 resilien*).ti,ab. (22)

6 (Paradigm adj8 resilien*).ti,ab. (120)

7 Checklist/ (3600)

8 Checklist.ti,ab. (26243)

9 (Tool* adj8 resilien*).ti,ab. (249)

10 (Framework adj8 resilien*).ti,ab. (934)

11 (Index adj8 resilien*).ti,ab. (119)

12 (Algorith* adj8 resilien*).ti,ab. (10)

13 (Indices adj8 resilien*).ti,ab. (45)

14 (Model* adj8 resilien*).ti,ab. (2050)

15 (Schem* adj8 resilien*).ti,ab. (47)

16 Safety II.ti,ab. (14)

17 Moments of resilience model.ti,ab. (0)

18 Integrated resilience framework.ti,ab. (0)

19 Concepts for applying resilience.ti,ab. (0)

20 WHO's six building blocks of a health system.ti,ab. (0)

21 The Hospital Safety Index.ti,ab. (1)

22 a capability-based organizational resilience framework.ti,ab. (0)

23 or/16-22 (15)

24 or/1-15 (137255)

25 23 or 24 (137268)

26 User involvement.ti,ab. (775)

27 IT solution*.ti,ab. (52)

28 (Robotic adj2 surgery).ti,ab. (28)

29 Telecare.ti,ab. (195)

30 (((DMAT or Disaster Medical Assist Team or DPAT or Disaster psychiatric assistance team or EMIS or Emergency Medical Information System or SPPED or Surveillance in Post-Extreme Emergencies) and Disasters) or Disaster Medicine* or Emergenc* Responder*).ti,ab. (193)

31 mHealth.ti,ab. (630)

32 (Knowledge adj3 shar*).ti,ab. (5169)

33 (Bed adj3 manag*).ti,ab. (45)

34 ((disaster adj (drill or plan or prepare* or manage)) or (emergenc* adj (drill or plan or prepare* or manage))).ti,ab. (827)

35 (capacit* adj3 plan*).ti,ab. (328)

36 (Discharge adj3 plan*).ti,ab. (1255)

37 Emergency shelter.ti,ab. (139)

38 ((Robust* adj5 health) or (Strengthen* adj5 health)).ti,ab. (1766)

39 (Learn* adj5 health*).ti,ab. (5520)

40 Reorganis*.ti,ab. (589)

41 (Flexib* adj5 health*).ti,ab. (526)

42 ((Capabl* adj5 health*) or (Capabil* adj5 health*)).ti,ab. (690)

43 (Resourceful* or (adapt* adj5 health)).ti,ab. (4083)

44 (Transform* adj5 health*).ti,ab. (1751)

45 (Recover* adj5 health*).ti,ab. (2730)

46 Resilien*.ti,ab. (31109)

47 exp emergency preparedness/ (1228)

48 (Innovat* adj5 health*).ti,ab. (2261)

49 Health system*.ti,ab. (11223)

50 Health Institution.ti,ab. (118)

51 Health governance.ti,ab. (112)

52 Health organisation.ti,ab. (509)

53 Global Health.ti,ab. (2772)

54 exp Health Care delivery/ (98551)

55 exp Global Health/ (2311)

56 (High income adj5 health*).ti,ab. (170)

57 (NHS or National Health Service).ti,ab. (6132)

58 Hospital*.ti,ab. (154120)

59 exp health care services/ (209553)

60 (health service* or health facilit*).ti,ab. (48336)

61 healthcare setting.ti,ab. (526)

62 or/38-48 (50860)

63 or/49-61 (359061)

64 or/26-37 (9603)

65 62 or 64 (59686)

66 25 and 63 and 65 (3593)

**Database: CINAHL (EBSCO Host)** Searched 14th September 2020

( ((program* or plan* or protocol or paradigm* or checklist or tool or framework or index or algorith* or indices or model* or schem*) NEAR/8 (resilien*)) ) or ( safety II or moments of resilience or integrated resilience or applying resilience or building blocks or hospital safety index or resilience framework ) AND ( user involvement or it solution or robotic near2 surgery or telecare or DMAT or DPAT or EMIS or SPPED or Disaster medicine or emergency responder* ) or ( mHealth or Knowledge near/3 shar* or bed near manage* ) or emergency shelter or (capacit* or discharge) near3 plan* or (disaster or emergenc*) near (drill or plan* or prepare or manage*) ) or ( health) N5 ( robust or learn* or reorganis* or flexib* or capab* or resource* or transform or recover or innovat* or high income ) AND ( health system or institution or governance or organisation or global health or ( delivery of healthcare or global health or health policy or NHS or National Health Service or hospital* or ( Health facility* or health service or Healthcare setting* )"

1812 references retrieved.

**Database: Cochrane Database of Systematic Reviews : 4^th^ Sept 2020**

Resilien*.ti,ab

7 reviews

2 protocols

**Database: CENTRAL: 1^st^ Sept 2020**

(resilien*) Near/5 (model*or plan* or program* or Checklist* or Tool* or Framework* or Index or Algorith* or Indices or Paradigm* or protocol*).ti,ab.

329 records retrieved.

**Database:Science Citation Index (WoS): Searched 7^th^ Sept 2020**

Strategy run on **title search only** using simplified terms :

#1 TI=(resilien*)

#2 “ TI =(program* or plan* or protocol or paradigm* or checklist or tool*or framework or index or algorithm* or indices or model* or scheme)

#3 #1 and #2

Refined by Publication Years **2015 – 2020**

1161 references retrieved

**Social Science Citation Index (WoS): Searched 7^th^ Sept 2020**

Strategy run on **title search only** using simplified terms :

#1 TI=(resilien*)

#2 “ TI =(program* or plan* or protocol or paradigm* or checklist or tool*or framework or index or algorithm* or indices or model* or scheme)

#3 #1 and #2

Refined by Publication Years **2015 – 2020**

754 references retrieved

**Databases: WoS Conference Proceedings Citation Index (both Science and Social Science)**

#1 TI=(resilien*)

#2 “ TI =(program* or plan* or protocol or paradigm* or checklist or tool*or framework or index or algorithm* or indices or model* or scheme)

#3 #1 and #2

ALL Years limit

1027 references retrieved.

**Other additional searches for grey literature were carried out in late early Sept.**

These used a selection of basic terms: (resilien* or disaster plan*) and health

The Joanna Briggs Institute: 233 references retrieved

ClinicalTrials.gov : 88 references retrieved

Open Grey: 44 references retrieved
