# Supplementary material for: Organizational resilience in healthcare: a review and descriptive narrative synthesis of approaches to resilience measurement and assessment in empirical studies
Source: BMC Health Serv Res. 2023 Apr 19;23:376. doi: 10.1186/s12913-023-09242-9 (PMC10113996; doi:10.1186/s12913-023-09242-9)
Supplement: Supplementary file 2 — Additional file 2: Appendix. Characteristics of included studies. [file 12913_2023_9242_MOESM2_ESM.docx]

Appendix. Characteristics of included studies.

| **Author/s, year** | **Study location** | **Purpose of the study** | **Disciplinary tradition or conceptual background** | **Format and description of resilience evaluation approach** | **Indicators or examples of indicators used** |
| --- | --- | --- | --- | --- | --- |
| Adini et al. 2012 | Israel | To investigate the preparedness of hospitals to a specific emergency scenario | Emergency preparedness | Structured evaluation tool  The approach involved a review of the standard operating procedures prior to a research site (hospital) visit by professional evaluators, followed by a site visit during which all other components of the emergency preparedness were observed and measured using the tool | 490 parameters encompassing various components of emergency preparedness under four categories: 1) Standard operating procedures; 2) Training and drills; 3) Knowledge of staff; (4) Infrastructure and equipment |
| Aladhrai et al. 2015 | Yemen | To evaluate the impact of the 2011 Yemeni revolution on hospital disaster preparedness two years apart | Disaster preparedness | WHO Hospital emergency response checklist  The approach involved self-evaluation by hospital representatives (mostly heads of emergency departments) two years apart. Hospitals’ affiliation and total number of beds were registered as background information | 92 items structured around 9 main components of emergency preparedness: (1) command and control, (2) communication, (3) safety and security, (4) triage, (5) surge capacity, (6) continuity of essential services, (7) human resources, (8) logistics and supply management, and (9) post-disaster recovery |
| Ambat and Navya, 2020 | India | To assess the preparedness level of hospitals against emerging infectious diseases | Emergency preparedness | Semi-structured questionnaire  Participants included were hospital administrators, doctors with experience in treating infectious diseases, nursing superintendent, microbiologist, and lab technician. The questionnaire  had a separate section of questions for all the participants, and the other section specific for administrative  staff, doctors and for laboratory personnel | No detailed information on indicators. Results reported based on the International Health Regulations core capacities under four domains: (1) prevent: (national legislation, policy, and financing, communication, and advocacy, antimicrobial resistance, biosafety and security); (2) detect (national laboratory system, surveillance, reporting, human resources); and (3) response (emergency preparedness, emergency response operations, linking public health and security authorities, medical countermeasures and deployment, and risk communication) |
| Ardalan et al. 2016 | Iran | To estimate hospital safety in the event of a disaster | Emergency and disaster preparedness | Farsi version of the WHO Hospital Safety Index (FHSI)  Diagnostic and validated instrument developed by the WHO that quantifies the structural safety and functional capacity of a hospital. Data collection was based on the self-assessments of the hospital disaster committees | 145 items covering structural, non-structural and emergency and disaster management areas, including general information about the hospital, recognizing risks, functional, non-structural,  and structural safety assessment |
| Awad and Cocchio, 2015 | US | To assess the preparedness of hospital pharmacies to provide pharmaceutical services in mass casualty scenarios | Emergency preparedness | An electronic cross-sectional survey  The survey was developed to assess the general knowledge of available resources and attitudes toward the preparedness of the pharmacies. The survey consisted of 12 questions formatted under the Likert scale. Sample included pharmacy departments at all identifiable acute-care hospitals | Knowledge of available resources, attitudes toward the preparedness of the pharmacy department, and expectations of government and national pharmacy organization recommendations to guide pharmacy preparedness |
| Bin Shalhoub et al. 2017 | Saudi Arabia | To identify and describe hospital disaster preparedness | Disaster preparedness | Questionnaire based on the WHO toolkit for assessing health-system capacity for crisis management and WHO hospital emergency response checklist  The questionnaire contained open and closed ended questions. Thirteen major private hospitals with more than 100 beds capacity were included in the study. Data was collected through an interview undertaken by a researcher. Participants included key informants in the hospital such as the hospital administrators, emergency managers and/ or a member of the hospital emergency preparedness and response committee | Preparedness of the hospital to mass-casualty incidents and the capacity for surge in emergency events |
| Brevard et al. 2008 | US | To compare disaster preparedness of a trauma centre with performance in an actual disaster | Disaster preparedness and response | Direct observation and recording of events from within the hospital before, during, and after the storm; retrospective review of the hospital master disaster plan and a survey of key staff present during and post-storm  The plan was retrospectively reviewed and compared with the actual events that occurred after the disaster; the survey was undertaken amongst key personnel from trauma and emergency medicine present during the disaster and assessed preparation for the storm, conditions within the facility, and areas of success and failure | Key areas related to preparation for the storm, conditions within the facility, and areas of success and failure |
| Cimellaro et al. 2010 | US | To quantify disaster resilience of hospital system | Disaster preparedness and recovery | A comprehensive model to quantify disaster resilience  The model combines loss estimation and recovery models and was applied to a network of hospitals in Memphis, Tennessee | The physical structural aspects and the organisational efficiency of hospitals |
| Cimellaro et al. 2017 | Italy | To develop a simplified model that could describe the ability of the hospital emergency department to provide service to all patients after a natural disaster or any other emergency | Resilience engineering | Discrete event simulation (DES) models  Emergency department of a hospital was modelled using a DES model. Different scenarios were considered assuming different patient arrival rates for different seismic intensities and different levels of functionality generated by incurring structural damage. Then, a simplified analytical model was proposed and tested to evaluate the patients waiting time without the need to run complex discrete event simulation models | Patient waiting time |
| Crowe at al. 2014 | UK | To assess resilience of different service reconfigurations | Operational research and disaster emergency | A model and prototype software tool  The approach involved development of a deterministic model to measure the impact of a given pattern of disruption to health-care resources and infrastructure in the first instance. The focus of the analysis was on the unmet demand that may occur as a result of disruption to resources or a changed pattern of demand, alone or in combination. A working version of a prototype software tool was then developed and populated using an illustrative example involving a hypothetical local health system under different service configurations. The model was finally run for three different service  configurations and each of the disruption levels (mild, moderate and severe) | Unmet demand as a result of disruption to resources or a changed pattern of demand, alone or in combination |
| Davis et al. 2020 | US | To quantitatively examine the extent to which different factors contribute to the resilience of hospital emergency departments during disaster-level overcrowding events | Emergency preparedness and emergency response | Modelling framework  The approach involved developing a modelling framework based on The National Emergency Department Overcrowding Scale (NEDOCS) score. The testing and analysis of the approach was based on data from actual disaster-level overcrowding events | Overcrowding as indicated by NEDOCS scores; the number of beds in use by hour; throughput timestamps (starting with arrival time to ED, time to ED bed, time to physician disposition, and time to floor bed); staffing schedule |
| Dewar et al. 2014 | Australia | To investigate acute hospital pandemic influenza preparedness | Disaster preparedness management | Questionnaire and semi-structured interviews  The approach included a questionnaire based on the hospital requirements described in the Victorian Health Management Plan for  Pandemic influenza. One response from each hospital was requested. The follow-up qualitative semi structured telephone interview covered  personal and professional experiences during the pandemic | Hospital planning information; workforce issues; and infrastructure and surge capacity |
| Djalali et al. 2013 | Iran  Sweden | To compare hospital preparedness, as measured by functional capacity, between Iran and Sweden | Emergency preparedness | The Functional Capacity module of the WHO Hospital Safety Index (HSI)  Hospital affiliation and size, and type of hazards, were compared between Iran and Sweden. The Functional Capacity module of the HSI was evaluated and calculated using the HSI checklist without modifications. The structural and non-structural elements, which are also part of the HSI according to WHO, were not included in this study. The module consisted of 61 elements that are grouped into five sub-modules | Hospital disaster committee and the emergency operations centre, operational plan for disasters, contingency plans for medical treatment in disasters, plans for the operation, preventive maintenance, and restoration of critical services, and availability of medicines, supplies, instruments, and other equipment for use in emergency |
| Gilson et al. 2017 | South Africa  Kenya | To explore the need for, and nature of, everyday resilience within health systems, considering the routine challenges they face, and the strategies employed to address them | Resilient health systems | Document reviews, in-depth interviews, group discussions and observations  Data collection process was designed to identify managerial responses to the challenges and assess whether or not they indicated everyday resilience using two conceptual lenses: vulnerability reduction programmes (absorptive, adaptive and transformative strategies) and three sets of latent capacities that enable organisations to problem-solve (cognitive capacities), generate a store of possible actions to draw on in response to future challenges (behavioural capacities) and access resources to respond to uncertain and surprising conditions (contextual capacities | Health managers’ and staff’ responses to chronic stresses |
| Gilson et al. 2020 | South Africa | To test an existing framework of everyday health system resilience (EHSR) in examining how a local health system responded to the chronic stress of large-scale organizational change | Resilient health systems | Observations, in-depth interviews, analysis of meeting minutes and secondary data. Analysis of data based on the everyday health system resilience (EHSR) framework  The study tested an existing framework of everyday health system resilience (EHSR) through tracking the stress experienced, the response strategies implemented and the consequences of large-scale organizational change over two years (2017–18) | Health managers’ and staff’ responses to chronic stresses |
| Higgins et al. 2004 | US | To assess preparedness for mass casualty events | Emergency preparedness | A survey instrument based on the Mass Casualty Disaster Plan Checklist and a brief supplemental bioterrorism preparedness survey based on a checklist developed for the Agency for Healthcare Research and Quality  The checklist was to convert it into a survey instrument. Some items on the checklist asked for a single answer to questions that contained 2 or more parts. These items were separated, unless the multiple parts were so closely related that a single response was appropriate. Some items of specific interest were added (e.g., respondents were asked how much their facility had spent on preparedness since 9/11). | 252 items grouped into: (1) appropriateness and adequacy of physical facilities; (2) organizational structures, (3) human resources; and (4) communication systems, and some items about facility’s spent on preparedness |
| Hosseini et al. 2019 | Iran | To rank hospitals based on the level of their preparedness for disasters | Disaster preparedness | Questionnaire based on the WHO Hospital Safety Index  (HSI) and the assessment of vulnerability elements at hospitals developed by Mulyasari et al. (2013)  The approach involved assessing the level of hospital preparedness in terms of structural, non-structural and functional preparedness, and human resources. The collected data were analysed using the TOPSIS technique | Structural preparedness (3 indicators), nonstructural preparedness (2 indicators), functional preparedness (13 indicators) and human resources (3 indicators) dimensions |
| Jacques et al. 2014 | New Zealand | To assess the loss of function of hospitals | Resilient systems | Damage and loss-of-function survey tool and fault-tree analysis  A survey tool was used to collect functional impact data from the hospitals. Using a fault-tree analysis method, descriptions of the loss of functionality of physical systems, the impact to healthcare services and support services, and the sharing of resources and transfer of patients in a hospital system were created. On a basis of this information, a resilience metric capturing the loss of hospital function was created | Management of medical surge: staff (availability of medical staff, support staff, and backup plans for staffing during an emergency), structure (damage to all physical space and support infrastructure), and stuff (loss  of supplies and damage to equipment) |
| Janati et al. 2017 | Iran | To evaluate emergency response by hospitals against potential disasters | Emergency management | WHO hospital emergency response checklist  The study population included all hospitals in Tabriz. The hospital emergency response checklist was used to collect data. Data entry and analysis were carried out using SPSS software (version 20) | 92 items structured around 9 main components of emergency preparedness: (1) command and control, (2) communication, (3) safety and security, (4) triage, (5) surge capacity, (6) continuity of essential services, (7) human  resources, (8) logistics and supply management, and (9) post-disaster recovery |
| Kagwanja et al. 2020 | Kenya | To examine the challenges experienced by the health system at a sub-national level using an ‘everyday resilience’ lens | Resilient health systems | Observations, reflective meetings and in-depth interviews with middle-level managers and peripheral facility managers  In-depth interviews were designed specifically to collect data for testing the conceptual framework (EHSR) and to supplement previous information from the longer-term health system governance work under the learning site. The interview guide included questions related to stressors experienced within the health system, how managers responded to these, who they worked with to respond to the stressors and what including who (within/outside the health system) enabled them to respond to these stressors | Shocks experienced by health managers (lack of clarity in roles and political interference; human and financial resource challenges; and reduced autonomy) and strategies in response to these stressors according to the framework (absorptive, adaptive and transformative strategies) |
| Khazaei Monfared et al. 2017 | Iran | To estimate hospital safety in the event of disasters | Emergency management | WHO Hospital Safety Index translated to Farsi and adapted to Iran’s context by Ardalan et al. (2016)  Diagnostic and validated instrument developed by the WHO that quantifies the structural safety and functional capacity of a hospital. The sample included six hospitals | 5 sections and 145 indices for the safety assessment of hospitals, including general information about the hospital, recognizing risks, functional, non-structural,  and structural safety assessment |
| Miniati and Iasio, 2012 | Italy | To evaluate the performance response of hospitals and systems of hospitals in terms of their capacity to cope during an earthquake disaster | Emergency management | The theory of complex systems analysis with the use of an input–output inoperability (Leontief) model and a rapid seismic vulnerability assessment with the field data collection using the WHO evaluation forms  The approach included: (1) development of an input–output Leontief model, (2) intrinsic hospital performance evaluation, (3) hospital response evaluation. The methodology was applied to rapid assessment of seismic risk in the Florence hospital system, which includes five health structures | Structural, non-structural and organizational factors such as staffing levels, emergency plans and redundancies in equipment |
| Mulyasari et al. 2013 | Japan | To assess hospitals’ earthquake preparedness | Disaster preparedness | Questionnaire based on the WHO Hospital Safety Index  (HSI) and the assessment of vulnerability elements at hospitals  The parameters and indicators for the questionnaire were developed by combining the safety approach of the HSI and the vulnerability elements of hospitals. Most of the questionnaires were distributed by mail and some by fax with a given timeframe (12–29 June 2012) for completion | Six parameters with 21 indicators from the “four pillars of hospital preparedness” including structural, non-structural, functional, and human resources (e.g., medical equipment for emergency medical service, medicine for emergency; tents for emergency medical service; earthquake- and fireproof building construction) |
| Naser et al. 2018 | Yemen | To assess the hospital preparedness against disasters | Disaster preparedness | WHO Hospital Emergency Response Checklist  The sample was all Aden City facilities that had a functional emergency department working 24/7 and inpatient care base. The total number of studied hospitals was 10.  They were invited by official letters enclosed with a copy of the evaluation checklist | 92 items structured around  9 main components of emergency preparedness: (1) command and control; (2) communication; (3) safety and security; (4) triage; (5) surge capacity; (6) continuity of essential services; (7) human resources; (8) logistics and management supply; and (9) post-disaster recovery |
| Paterson et al. 2014 | Canada | To assess the resiliency of healthcare facility to climate change impact | Emergency management, facilities management and health care services | A climate change resiliency assessment toolkit  The toolkit consists of a checklist for officials who work in areas of emergency management, facilities management and health care services and supply chain management, a facilitator’s guide for administering the checklist, and a resource guidebook to inform adaptation. The checklist consisted of 82 questions and was reviewed and revised based on input from the expert advisory committee before being further tested at health care facilities | Four broad areas: general information; assessing climate-related risks; risk management and building capacity to adapt to climate change |
| Prateepko and Chongsuvivatwong, 2012 | Thailand | To assess and document patterns of basic preparedness for a threat of an influenza pandemic | Emergency preparedness | A checklist for health care facilities  The checklist was designed and developed based on the WHO checklist for influenza pandemic preparedness planning and the preparedness checklist for long-term care facilities, other international infection control and preparedness checklists and Thailand’s influenza pandemic preparedness plans. Key staffs were asked to complete the checklist. Descriptive statistics were used to present the results | Facility access plan, surveillance, infection control, risk communication and health information dissemination, and health alert network and information technology |
| Rios et al. 2021 | Puerto Rico | To assess hospital and health system resilience after Hurricane Maria | Resilient health systems | Interviews with healthcare professionals analysed using Kruk et al.’s resilience framework  The interview guide was designed to prompt discussion about the strengths and weaknesses contributing to the health outcomes. The data were analysed through the lens of the five resilience domains.Y hirteen Emergency Medicine Physicians, Family Medicine Physicians, and Hospital Administrators in a University of Puerto Rico (UPR) Community Hospital were interviewed as part of our study | Five foundational components of Kruk’s resilience framework: (1) Aware, (2) Diverse, (3) Self-Regulating, (4) Integrated, and (5) Adaptive |
| Sharma and Sharma 2020 | India | To understand hospital preparedness and resilience at times of health emergencies, including a pandemic, the most current one being COVID-19 | Emergency preparedness | A semi-structured online questionnaire and available published and unpublished data for situation analysis  The questionnaire had 24 questions and was sent to those who had attended a training programme on disaster preparedness in hospitals. Additionally, available published and unpublished data for situation analysis was explored. Data analysis was conducted using IBM SPSS version 22 | No detailed information on indicators. Results organised under the following preparedness areas: public health emergency preparedness; training; capacity to deal with an emergency; and prior experience in managing an emergency |
| Shirali et al. 2016 | Iran | To present and test a new framework for assessing the crisis management based on resilience principles in hospital infrastructure | Resilience engineering | Questionnaire based on the seven dimensions of resilience engineering  The questionnaire consisted of 44 questions. The themes of the questions were designed in a way to measure the crisis management dimensions in four phases, i.e., prevention, preparedness, response, and recovery. 310 staff (nurses and managers) of eight hospitals in Iran completed the questionnaire | Seven dimensions of  resilience engineering: top management commitment,  just culture, learning culture, opacity, preparedness, awareness, and flexibility |
| Sobhani et al. 2014 | Iran | To determine the preparedness level of hospitals against natural disasters | Emergency preparedness | A standard checklist  The checklist comprised 220 items in 10 areas and was completed for each hospital using observations and interviews. The study was conducted in all the 9 hospitals in Bandar Abbas, Iran, during 2012. The checklist was completed for each hospital using observations and interviews. SPSS 16.0 was used to analyse the data | 220 items in 10 areas: emergency services (30 items), reception (24 items), evacuation (30 items), traffic (15 items), communication (16 items), security (17 items),  training (17 items), logistics (28 items), human resources  (21 items), and commanding and management (22 items) |
| Ten Eyck, 2008 | US | To assess surge response plans hospitals in the study | Emergency management | Standardized data form evaluating surge response plans  Using a standardized data form, surge response plans for each hospital were assessed. The cumulative results were compared to the demand projected for an avian influenza pandemic using the CDC's FluAid and FluSurge models | Hospital surge capacity |
| Traub et al. 2007 | Australia | To measure physical assets in hospitals required for the management of mass casualties as a result of terrorism or natural disasters | Emergency management | Hospital surge capacity survey  The survey was designed to collect data on hospital surge capacity measures. Sample included all emergency department directors of Australasian College for Emergency Medicine (ACEM)-accredited hospitals, as well as private and non-ACEM accredited emergency departments staffed by ACEM Fellows in metropolitan Sydney | Surge capacity measures: numbers of operating theatres, intensive care unit (ICU) beds and x-ray machines; state of preparedness using benchmarks |
| Ul-Haq et al. 2019 | Pakistan | To assess the status of preparedness of health-system components for crisis management | Disaster preparedness | WHO Toolkit for assessing health-system capacity for crisis management  The toolkit consisted of a “User manual” and the “Assessment form”. It was structured using the WHO health systems framework - subcategorized into 16 key components and 51 essential attributes. Preparedness at the district management as well as secondary and tertiary health care facilities level | 16 key components and 51 essential attributes (229 indicators) based on the WHO health systems framework divided into nine key components: command and control; communication, safety and security, triage, surge capacity, continuity of essential services, human resources, logistics and supply management, post-disaster recovery |
| Yavari et al. 2010 | US | To develop and demonstrate a model for estimating the post-disaster functionality of hospitals | Emergency response and management | Predictive model for estimating the post-disaster ability to provide services  Observational data on the performance of hospitals in past earthquakes, identifying key factors that have been found to affect hospital functionality were summarised. The predictive model of hospital functionality based on statistical analysis of post-earthquake inspection data was then developed. As a case study, the methodology was utilized to predict the functionality of hospitals in Los Angeles County for two earthquake scenarios | Functionality of hospital:  structural, nonstructural, lifelines, and personnel |
| Zhong et al. 2014 | China | To assess the overall ability of hospitals to cope with disasters | Resilience engineering | A framework and derived questionnaire for measuring hospital disaster resilience  An evaluation framework for assessing hospital resilience was initially proposed through a systematic literature review and Modified-Delphi consultation. Within this framework, eight key domains were identified that reflected the level of hospital resilience. Then, based on the preliminary framework, a hospital survey questionnaire was designed to collect the data. The data from the returned questionnaires were analysed using factor analysis to extract component factors | Emergency medical response capability, disaster management mechanisms, hospital infrastructural safety, and disaster resource |
